# Interacting Abiotic Factors Affect Growth and Aflatoxin B_1_ Production Profiles of *Aspergillus flavus* Strains on Pistachio-Based Matrices and Pistachio Nuts

**DOI:** 10.3389/fmicb.2020.624007

**Published:** 2021-01-20

**Authors:** Alaa Baazeem, Esther Garcia-Cela, Angel Medina, Naresh Magan

**Affiliations:** ^1^Department of Biology, College of Science, Taif University, Taif, Saudi Arabia; ^2^Applied Mycology Group, Environment and AgriFood Theme, Cranfield University, Cranfield, United Kingdom

**Keywords:** water relations, growth, mycotoxins, boundary conditions, optimum conditions, aflatoxins, pistachios

## Abstract

Pistachio nuts are an economically important commodity produced by many countries. They can be colonized by mycotoxigenic fungi, especially *Aspergillus flavus*, resulting in contamination with *aflatoxins (AFs)*, especially aflatoxin B_1_ (AFB_1_), a Class 1a carcinogen. The objectives were to examine the effect of interactions between the two key abiotic factors, temperature and water activity (a_*w*_) on (a) *in vitro* growth and AFB_1_ production by four strains of *A. flavus* isolated from pistachio nuts, on a milled pistachio nut medium modified ionically (NaCl) and non-ionically (glycerol) in the range 20–35°C and 0.995–0.85 a_*w*_, (b) colonization of layers of raw pistachio nuts stored at different interacting temperature x a_*w*_ conditions and on relative AFB_1_ production and (c) develop models to produce contour maps of the optimal and marginal boundary conditions for growth and AFB_1_ production by up to 4 strains of this species. On pistachio nut-based media, optimum growth of four strains of *A. flavus* was at 0.98–0.95 a_*w*_ and 30–35°C. Optimum AFB_1_ production was at 30–35°C and 0.98 a_*w*_. No significant differences in growth was found on ionic and non-ionically modified media. Colonization of layers of raw pistachio nuts was slower and contamination with AFB_1_ significantly less than in *in vitro* studies. Contour maps based on the pooled data for up to four strains (*in vitro*, *in situ*) showed the optimum and marginal conditions for growth and AFB_1_ production. These data can be used to identify those conditions which represent a high, intermediate or low risk of colonization and AFB_1_ contamination in the pistachio nut processing chain. These results are discussed in the context of the development of appropriate intervention strategies to minimize AFB_1_ contamination of this economically important commodity.

## Introduction

Pistachio nuts (*Pistacia vera* L.) are an economically important tree nut crop for many countries including the United States, Iran, Southern Europe and China. Of these, Iran and the United States are the major producers and exporters of pistachio nuts (FAOSTAT, 2020). Pistachio nut quality and safety has thus become an important factor because they are prone to infection by species from the *Aspergillus* section *Flavi* group, especially *A. flavus* pre-harvest, if the shells become split early, or due to poor post-harvest drying or storage practices (Kaminiaris et al., 2020). This can result in significant contamination with aflatoxins (AFs). This has resulted in many countries including the EU having strict legislative limits on the maximum contamination levels with aflatoxin B_1_ (AFB_1_) or total AFs allowable. Indeed, for a few years Iranian pistachio nuts were excluded for importation into EU countries because of batches consistently exceeding the prevailing legislative limits for AFs (Bui-Klimke et al., 2014). This required action, especially in the harvest and post-harvest phases of the chain to be improved to reduce the contamination levels with these carcinogenic mycotoxins to levels to meet the legislative maximum limits.

Pistachio nuts in various processed forms are very popular in the Middle East and have been imported into most countries including the Kingdom of Saudi Arabia (KSA) (Mozaffari, 2012). Examination of a range of such pistachio nuts from markets in KSA originating from various producers and processors showed that *Aspergillus* section *Flavi* strains was present in many of the samples as well as contamination with AFs (Nawar, 2008; Baazeem, 2018). In addition, in other countries including the United States, Iran, Spain and Greece *A. flavus* has often been the predominant species encountered in pistachio nuts (Ciegler, 1972; Mojtahedi et al., 1979; Fernane et al., 2010a,b; Varga et al., 2011).

There is interest in a better understanding of the key abiotic factors which affect infection and colonization of pistachio nuts pre-harvest during early shell splitting, and post-harvest during drying and storage (Georgiadou et al., 2012). For example, drought stress can lead to increased shell spliting and allow inoculum of *A. flavus* to colonize the nuts and contaminate them with AFs (Sommer et al., 1986; Doster and Michailides, 1995; Kaminiaris et al., 2020). In addition, harvested pistachio nuts are very hygroscopic, because of their high lipid content, and can re-absorb moisture from the atmosphere, especially during storage or transport that increases the water availability to levels that can become conducive to growth of such xerophilic fungi and AFs contamination. Generally, to maintain and conserve quality, pistachios need to be dried to <6–7% moisture content (= ≤0.70 water activity, a_*w*_) and then stored without an increase in water availability during storage or transport and processing phases to avoid the risk of *A. flavus* colonization and contamination with AFs.

It is thus important to understand the relationship between interacting abiotic factors, especially of a_*w*_ and temperature on the colonization by *A. flavus* and contamination of the pistachio nuts with AFB_1_. Some studies have examined germination, growth and AFB_1_ production by single strains of *A. flavus* isolated in Spain on pistachio nut-based media and on stored pistachios (Marin et al., 2012; Aldars-García et al., 2015). Interestingly, having tested different storage m.c. conditions the studies suggested that storage at ≤10% was necessary to minimize AFB_1_ contamination of pistachios to below the EU legislative levels. However, comparisons were not made between different strains of *A. flavus* isolated from raw or roasted, salted nuts. While there is a significant amount of data on *A. flavus* ecology and water relations in maize and peanuts, less is available on pistachio nuts (Giorni et al., 2008; Abdel-Hadi et al., 2012).

The objectives of this study were to examine the effect of interacting abiotic factors of a_*w*_ x temperature on growth and AFB_1_ production by four different *A. flavus* strains isolated from pistachio nuts, on (a) pistachio nut-based media and (b) in stored raw pistachio nuts. The data was used to build boundary models for growth and AFB_1_ production to identify the overall optimal and marginal conditions which represent the highest and lowest risks for growth and toxin contamination.

## Materials and Methods

### *Aspergillus flavus* Strains

Four strains of *A. flavus* isolated from pistachio nut samples were molecularly identified using two sets of primers (ITS 1/2, ITS 3/4; Supplementary Table 1). They were coded as AB3, AB4, AB5, and AB10 and identification was further confirmed by comparison with a characterized type strain of *A. flavus* (NRRL 3357) from the Agricultural Research Service Culture Collection (United States), isolated from maize grain (Supplementary Table 2). They were all aflatoxin B_1_ producers.

### Preparation of *in vitro* Growth Media

The growth medium used was a 3% milled pistachio nut agar (PNA). To prepare this medium, raw unsalted pistachio nuts were milled to a powder in a homogeniser. The milled pistachio powder was then sieved to obtain a uniform size. Thirty grams (30 g) of the pistachio powder and 20 g technical agar (Thermo Fisher Scientific Oxoid Ltd., Basingstoke, Hampshire, United Kingdom) and 0.05 g chloramphenicol (antibacterial agent) was added to 1 L distilled water for the basal medium (0.99 a_*w*_). The a_*w*_ was modified using either the ionic solute NaCl, or the non-ionic solute glycerol, to 0.85, 0.88, 0.90, 0.93, 0.95, 0.98, and 0.995 a_*w*_ (Lang, 1967; Dallyn and Fox, 1980). For the ionic solute NaCl concentrations were directly added to the PNA media prior to autoclaving. For glycerol-amended media, the glycerol/water mixtures were made up, shaken vigorously and then added like water, to the pistachio nut flour and agar. The treatments were then autoclaved at 121°C for 15 min. After autoclaving, the PNA treatments were cooled, mixed thoroughly, poured into 9 cm sterile Petri plates (17.5–20 ml per plate) and allowed to completely cool and solidify. The a_*w*_ was measured using an Aqualab 4TE (Decagon Instruments). The media treatments were enclosed in separate closed polyethylene bags and stored at 4°C until use.

### Fungal Inoculation and Growth Rate Measurement of *Aspergillus flavus* Isolates on Milled Pistachio Nut-Based Agar Media

The PNA plates were equilibrated at 25°C and then centrally inoculated with the strains of *A. flavus* (4 isolates). Inoculum consisted of a conidial spore suspension of each strain made from fresh 5–7 day old growing cultures on PNA at 25°C. The culture surface was gently scraped with a sterile loop and conidia were transferred into sterile 25 ml Universal glass tubes containing 10 ml sterile water +0.1% Tween 80 solution (Tween 80, ACROS organics). The concentration of the spore suspension was determined using a haemocytometer (Olympus BX40 microscope, Microoptical Co.; slide Marienfeld superior, Germany; microscope glass cover slips, No 3, 18 × 18 mm, Chance proper LTD, United Kingdom) and adjusted by dilution with sterile water to 10^6^ spores ml^–1^**.** The treatments and replicates were centrally inoculated with 10 μL of the spore suspension. The inoculated treatments and replicates were incubated at 20, 25, 30, and 35°C. Growth was assessed by measuring colony diameters of *A. flavus* every day for up to 10 days. Measurements of three replicates of each treatment were recorded on an excel sheet. The radial growth rates (mm/d) were obtained by computing the radial extension rate into the *y = mx* + *c* equation, where *y* was the radius (mm), *x* was day (d) and *m* was the growth rate (mm/d). *c* was set to intercept at 0 by assuming that at day 0, the radius was 0.

### Colonization of Pistachio Nuts Under Different Temperature × Water Activity Conditions by Strains of *Aspergillus flavus*

*Moisture adsorption curve for pistachio nuts:* The pistachio nuts used in this study were gamma irradiated at 12–15 kGys (Synergy Health Sterilization United Kingdom Ltd., Swindon, Wiltshire, United Kingdom) to remove any resident microbiota present. To accurately modifiy the a_*w*_ of these raw pistachio nuts, a water adsorption curve was developed. The relationship between added water and a_*w*_ was obtained by adding known amounts of water to 5 g sub-samples of raw pistachio nuts in 25 mL Universal glass bottles. These were shaken, sealed and left at 4°C overnight. After thorough mixing and equilibration at 25°C, the a_*w*_ was determined for each sub-sample using the Aqualab 4TE water activity meter (Aqualab 4TE; Decagon Devices, Inc., Pullman, WA, United States). This water adsorption curve was subsequently used to accurately determine the amounts of water necessary to obtain the target a_*w*_ levels in the experimental studies. We used the following modified a_*w*_ levels of the raw pistachio nuts: 0.88, 0.90, 0.93, 0.95, 0.98, and 0.995 (= 9.5–10, 11–12, 13–14, 18–19, 26–27, and 32–35% m.c.).

### Fungal Inoculation and Growth Rate Measurement of *Aspergillus flavus* Isolates on Pistachio Nuts

Single layers of the raw pistachio nuts were spread into 9 cm sterile Petri plates in a flow bench. These were centrally inoculated with the individual strain (AB3 and AB10) of *A. flavus* using an agar plug (4 mm diameter) of germinating spores of each strain. The experiment was carried out with three replicates per treatment at 20, 25, 30, and 35°C and carried out twice. The Petri plates were placed in large surface-sterilized plastic chambers where the ERH was maintained with glycerol/water solutions (approx. 2 × 750 mL) to the target a_*w*_ levels of the nut treatments. The colonization rates were measured on a daily basis for up to 10 day. The glycerol/water solutions were replaced after 5 days with fresh solutions.

### Aflatoxin B_1_ Quantification

*Preparation of aflatoxin standards:* A 200 μL stock solution of aflatoxins (B_1_, B_2_, G_1_, and G_2_) standard in methanol containing 250 ng AFB_1_ was prepared and pipetted into 2 mL Eppendorf tubes for overnight evaporation until dryness in a fume hood similar to the samples.

### *In vitro* Aflatoxin B_1_ Analyses

#### Colony Extraction

Initially agar plugs were cut out across the diameter of colonies using a surface sterilized 4 mm diameter cork borer (approx. 4–6). The agar plugs were placed in pre-weighed 2 mL Eppendorf tube and weighed again. Five-hundred μL of high performance liquid chromatography (HPLC)-grade chloroform was added to the tubes and shaken for 30 min using a KS 501 digital orbital shaker (IKA^®^ Werke GmbH & Co., KG, Germany). The chloroform extract was transferred to a new Eppendorf tube, dried gently under air for derivatisation.

#### Derivatisation of Aflatoxin B_1_ Extract

Derivatisation of the AFB_1_ extract was performed according to the AAOC method (Kok, 1994). First, 200 μL hexane was added to the tube followed by 50 μL of triflouroacetic acid. The mixture was vortexed for 30 s and left for 5 min. A mixture of water:acetonitrile (9:1) was then added to the tube, and vortexed for 30 s and left for 10 min to allow for separation of the layers. Then the aqueous layer was filtered using a syringe nylon filter (13 mm × 0.22 μm; Jaytee Biosciences Ltd., United Kingdom) into amber salinized 2 mL HPLC vials (Agilent, United States) before HPLC analysis. All analytical reagents used were HPLC-grade.

#### Quantification of Aflatoxin B_1_ With High Performance Liquid Chromatography

A reverse-phase HPLC with fluorescence detection was used to confirm the identity and quantify AFB_1_. An Agilent 1200 series HPLC system was used for the analysis. It consisted of an in-line degasser, auto sampler, binary pump and a fluorescence detector (excitation and emission wavelengths of 360 and 440 nm, respectively). Separation was achieved using a C_18_ column (Phenomenex Gemini; 150 × 4.6, 3 μm particle size; Phenomenex, United States) with a Phenomenex Gemini C_18_ 3 mm, 3 μm guard cartridge. Isocratic elution with methanol:water:acetonitrile (30:60:10, v/v/v) as the mobile phase was performed at a flow rate of 1.0 mL/min. The injection volume was 20 μL. A set of standards was injected (1–5 ng AFB_1_, AFB_2_, AFG_1_, and AFG_2_ per injection) and standard curves were generated by plotting the area underneath the peaks against the amounts of AFB_1_ standard injected.

### Quantification of Aflatoxin B_1_ in Pistachio Nuts

The dried pistachio nut samples (25 g) were ground and weighed. The background aflatoxin B_1_ levels in the nuts used in the experiments was 0.015 ng/g. This was taken into account in the final quantification of the results. Acetonitrile/water 60/40 (100 mL) was used as an extraction solvent. The mixture was blended for 3 min and the extract filtered into a smaller sample container. PBS buffer was used for sample dilution, then the diluted extract was passed through an Immunoaffinity Column (IAC; AflaStar^TM^; Romer Labs, Austria) with a flow rate between 1 and 3 mL/min. The column was rinsed with 2 × 10 mL sterile distilled water. HPLC-grade methanol (1.5–3 mL) was then applied to the column and the eluent was collected in a new vial and left to dry overnight before the derivatisation step as detailed previously.

### Statistical Analysis

All experiments were carried out twice. Three replicates per treatment were used in all experiments. Analysis of Variance (ANOVA) was applied to analyze the variation of means with 95% confidence interval. Normal distribution of data was checked by the normality test Kolmogorov–Smirnov using Minitab statistical software. Fisher’s Least Significant Difference (LSD) was used to identify differences between the means with *p* < 0.05 as the criterion for significant difference using the same statistical software.

Individual fungal growth rate (mm/day) and AFB_1_ production (ng/mL) as well as the pooled data sets for the different strains on PNA media or on pistachio nuts were fitted using non-linear regression with the software package Statgraphics Centurion 18

$$\mathrm{Radial}\,\mathrm{fungal}\,\mathrm{growth}\,\left(\frac{m\,m}{d\,a\,y}\right)\,\mathrm{or}\,{\mathrm{AFB}}_{1}\,\,\left(\frac{\text{ng}}{\text{mL}}\right)\;=a+b\cdot T+c\cdot a_{w}+d\cdot T^{2}+e\cdot a_{w}^{2}+f\cdot T\cdot a_{w}.$$

Contour maps were built using data from the predicted formula in Sigma Plot 14.

## Results

### Effect of Temperature × Water Activity on Growth and Contour Maps for Optimum and Marginal Conditions for *Aspergillus flavus* Strains on Milled Pistachio Nut-Based Media Modified Ionically or Non-ionically

**FIGURE 1:**
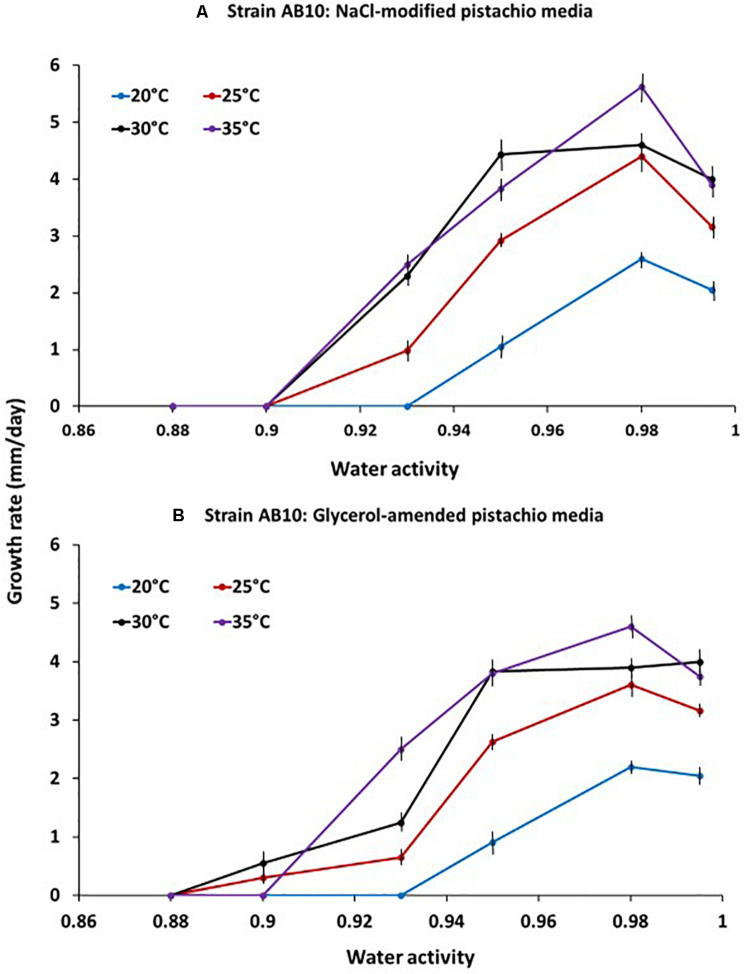
Effect of temperature x water activity on growth of *A. flavus* strain AB10 on a milled pistachio nut agar medium modified with either **(A)** NaCl or **(B)** glycerol. Based on growth over a 10 days incubation period. Bars represent the Standard Error of the means.

An example of the effect of temperature x a_*w*_ on the relative growth rates of *A. flavus* strain AB10 on the PNA solute modified media is shown in Figure 1. Optimum conditions for growth were between 30–35°C and 0.95–0.98 a_*w*_. Growth rates were similar on both NaCl- and glycerol-amended PNA media although the strain was slightly more sensitive to the ionic solute at marginal conditions for growth (e.g., 0.90 a_*w*_).

**FIGURE 2:**
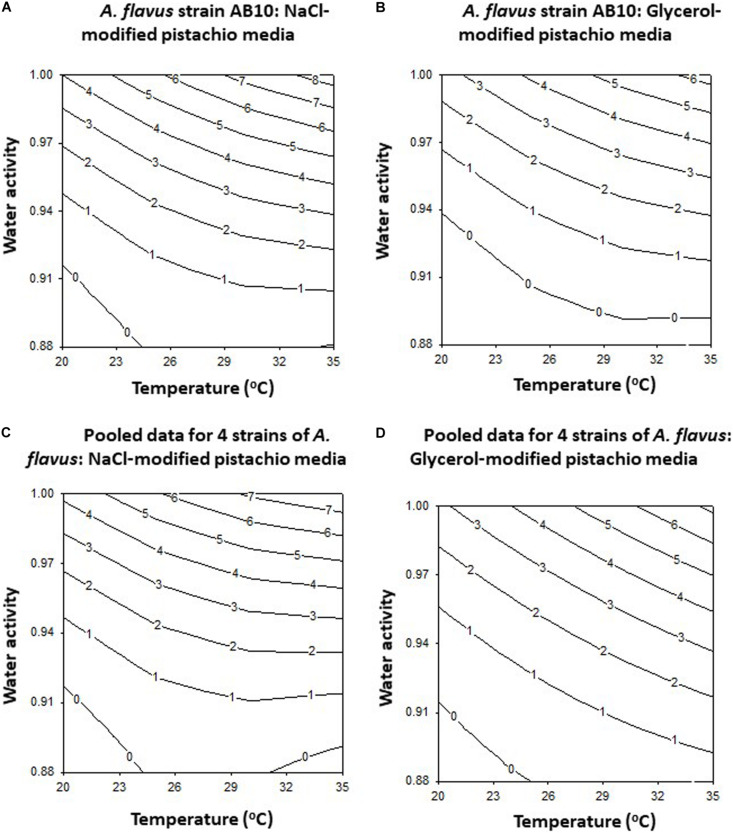
The contour maps for growth of *A. flavus* strain AB10 on **(A)** NaCl and **(B)** glycerol-amended pistachio nut agar media. The pooled data for 4 strains (AB3, AB4, AB5, and AB10) is shown for solute-modified media for **(C)** NaCl and **(D)** glycerol.

The contour maps developed for this strain (AB10) and for the pooled data for the four strains examined (AB3, AB4, AB5, and AB10) are shown in Figure 2. This clearly shows that on both solute modified media optimum growth rates are at >30°C and >0.98 a_*w*_ for strain AB10 (Figures 2A,B) and also for the pooled data for the 4 strains examined (Figures 2C,D). Boundary conditions were close to 0.90–0.91 a_*w*_ over the temperature range examined for both types of solute-modified PNA media.

### Temperature × Water Activity Abiotic Factors on Aflatoxin B_1_ Production by Strains of *Aspergillus flavus* on Milled Pistachio Nut-Based Media Modified Ionically or Non-ionically

**FIGURE 3:**
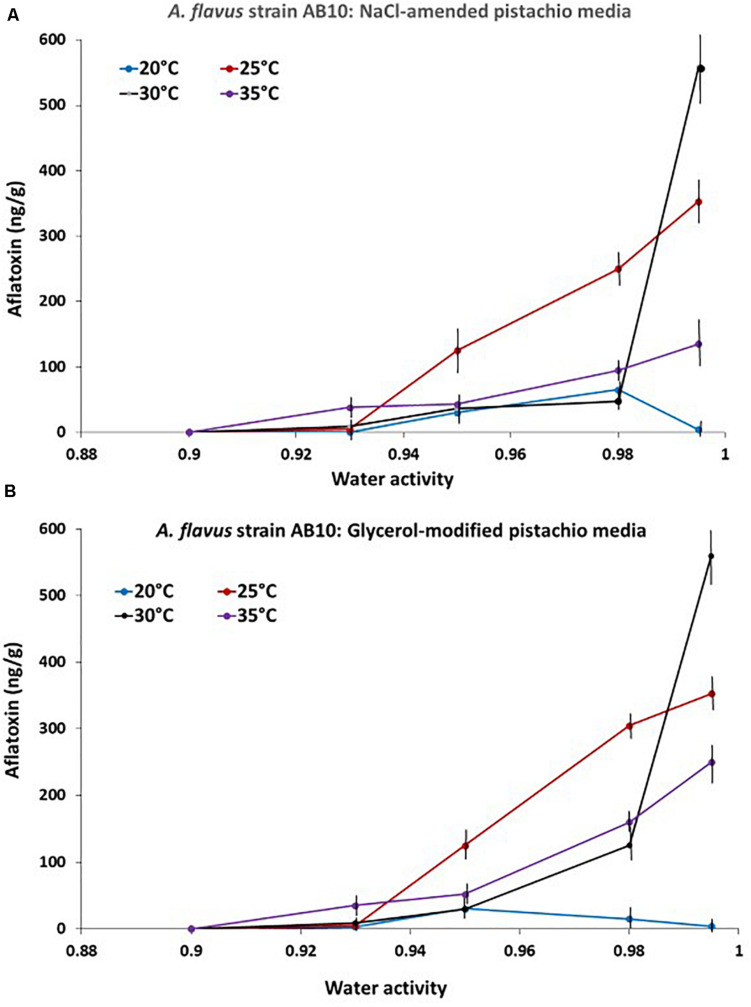
Effect of temperature x water activity on aflatoxin B_1_ production by *A. flavus* strain AB10 on a milled pistachio nut agar medium modified with either **(A)** NaCl or **(B)** glycerol. Quantification was done after 10 days incubation. Bars represent the Standard Error of the means.

The relative production of AFB_1_ by strain AB10 of *A. flavus* on the PNA media modified with NaCl or glycerol as an example is presented in Figure 3. This clearly shows that maximum AFB_1_ was produced at 30°C and 0.99 a_*w*_. No toxin was produced at 0.90 a_*w*_ over the time periods of our experiments at the temperatures examined. This was followed by production over a wider a_*w*_ range at 25°C. Interestingly less AFB_1_ was produced at 35°C despite the faster colonization rate observed on PNA-media modified with NaCl or glycerol. In addition at a marginal temperature for growth (20°C) more AFB_1_ was produced at 0.95 and 0.98 a_*w*_.

**FIGURE 4:**
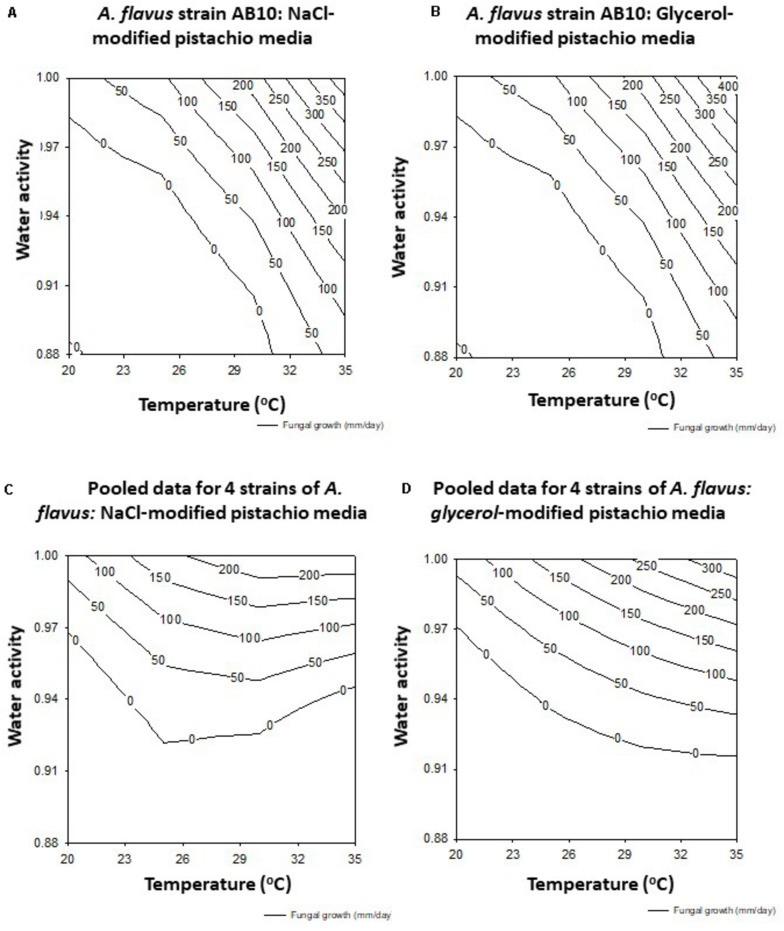
Contour maps for aflatoxin B_1_ production (ng/g) by strain AB10 on milled pistachio nut agar modified with **(A)** NaCl) or **(B)** glycerol. The pooled data for four strains (AB3, AB4, AB5, AB10) on the same media is shown in **(C,D)** respectively.

The data was used to develop the models for the contour maps for optimum and marginal AFB_1_ production conditions for the 4 strains together on both solute-modified PNA media (Figures 4A,B). This shows that the predicted AFB_1_ production would be optimum over a wide temperature range of 25–35°C at >0.98 a_*w*_.

### Effect of Temperature × Water Activity on Colonization Rates and Contour Maps of Growth Profiles for the *Aspergillus flavus* Strains on Raw Pistachio Nuts

**FIGURE 5:**
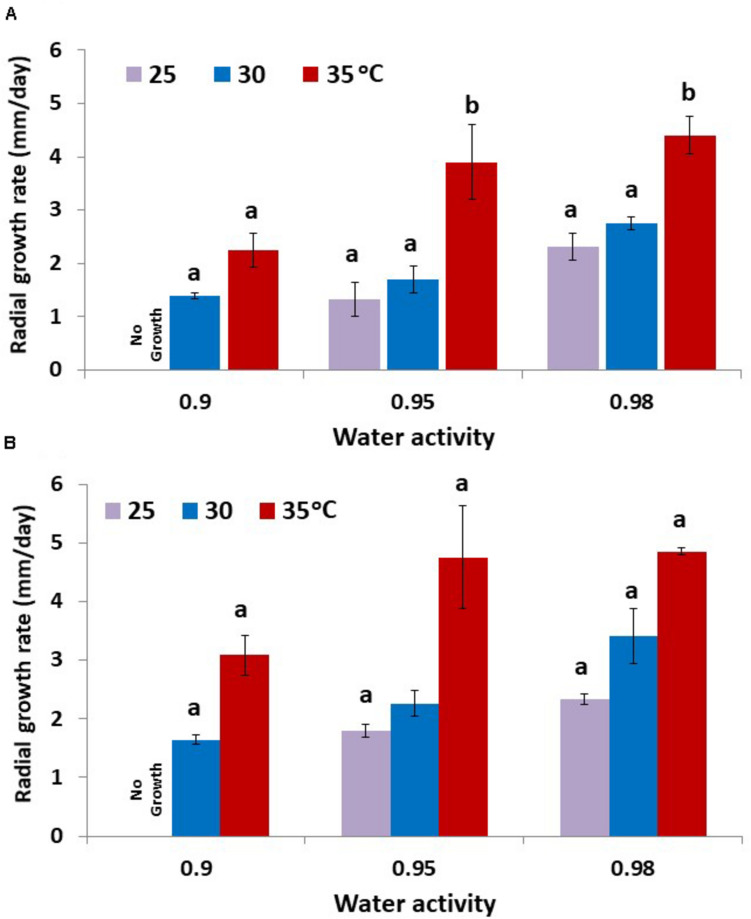
Colonization of layers of raw pistachio nuts by *A. flavus* strain **(A)** AB3 and **(B)** AB10 at different water activity levels and different temperatures. Data are means of three replicates. Bars represent the Standard Error of the means. Different letters indicate significant differences (*p* < 0.05).

The colonization rates of layers of raw pistachio nuts by strains AB3 and AB10 were examined (Figure 5). Both strains showed similar behavior with growth rate increasing as temperature or a_*w*_ were increased. There was no growth observed at 0.90 a_*w*_ when incubated at 25°C. Strain AB10 showed slightly faster colonization rates than strain AB3. There was no significant difference between the growth rate of the two strains at 25 and 30°C and 0.95 and 0.98 a_*w*_. However, at 35°C growth was almost double that at 30°C at all a_*w*_ levels tested for both strains.

**FIGURE 6:**
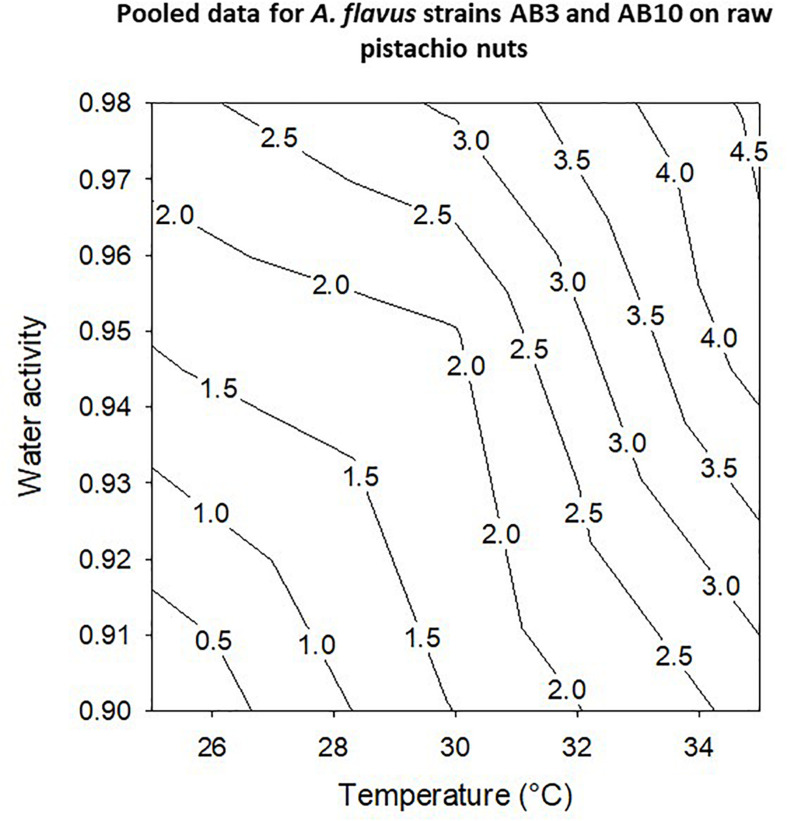
Contour maps for pooled data for growth of *A. flavus* strains AB3 and AB10 on layers of raw pistachio nuts using growth rates over periods of 10 days.

The contour maps show the profiles for optimum and marginal conditions of colonization of layers of pistachio nuts (Figure 6). Colonization rates were best at 30–35°C and over a wide range of a_*w*_ conditions with maximum at >0.95–0.98 a_*w*_ for the pooled data for the two strains (AB3 and AB10; Figure 6).

### Effect of Temperature × Water activity on Aflatoxin B_1_ (ng/g) Production of *Aspergillus flavus* Strains and Contour Maps on Raw Pistachio Nuts

**FIGURE 7:**
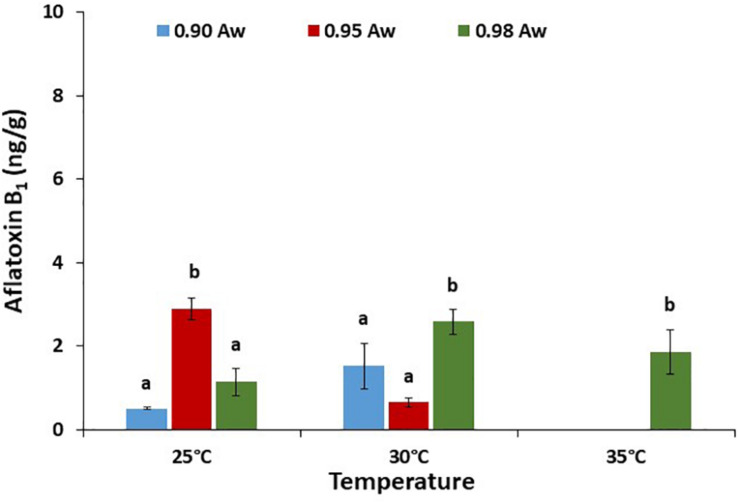
Aflatoxin B_1_ production (ng/g) by *A. flavus* strain AB10 grown on a layer of raw pistachio nuts at different water activities and temperatures. Data are means of three replicates. Bars represent Standard Error of the means. Different letters indicate significant difference (*p* < 0.05).

The effect of a_*w*_ × temperature on AFB_1_ production by the AB10 strain on raw pistachio nuts is presented in Figure 7. Overall, the production of AFB_1_ on pistachio nuts was at least 10–15x lower than that produced on PNA media. AFB_1_ production was significantly higher at 0.98 a_*w*_ and 30 and 35°C when compared with the other conditions examined. No AFB_1_ was detected at 35°C except at 0.98 a_*w*_ for strain AB10.

**FIGURE 8:**
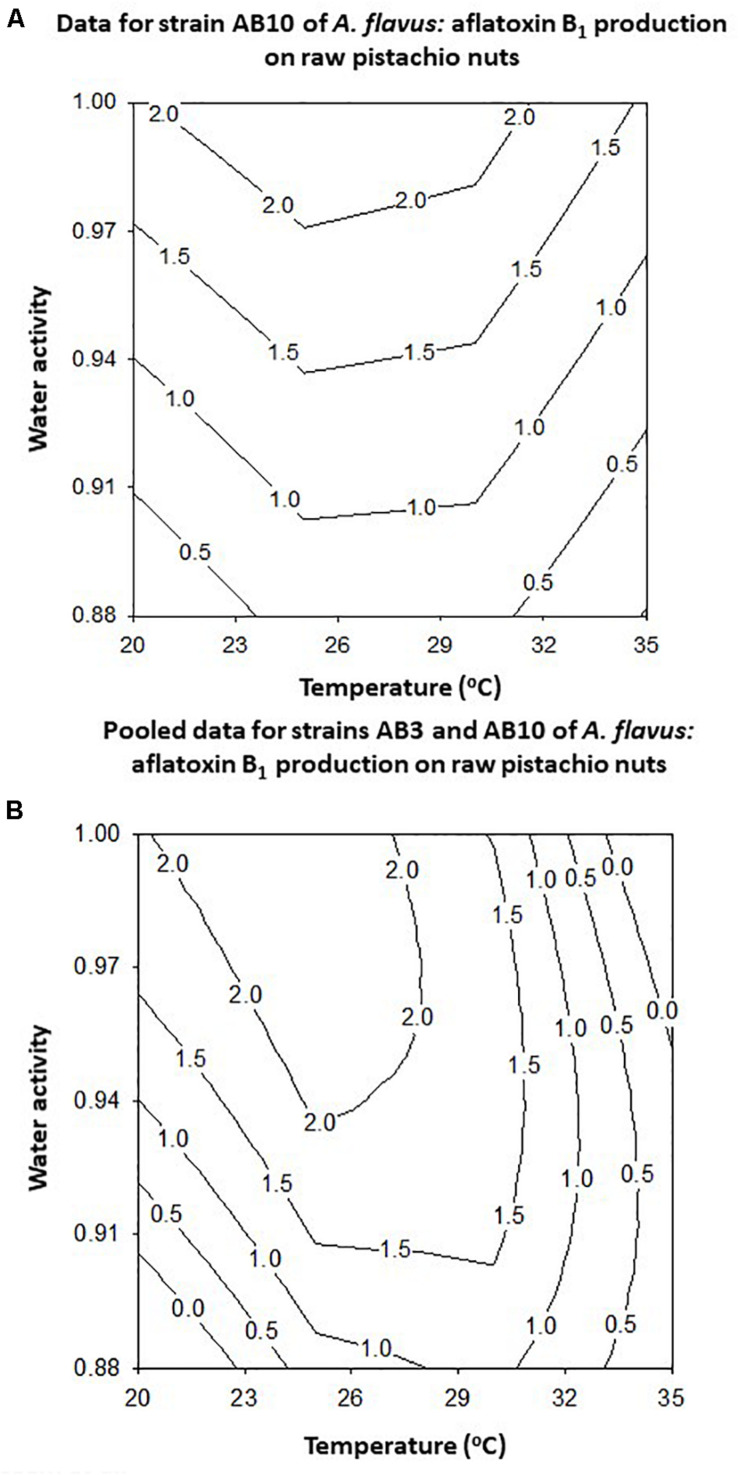
Contour maps for the effect of temperature x water activity on aflatoxin B_1_ by *A. flavus* strain AB10 **(A)** and for the pooled data for strains AB3 and AB10 **(B)** produced after 10 days incubation on layers of raw pistachio nuts.

The modeling of the contour maps for joining similar production levels across the range of conditions examined showed that for strain AB10 the optimum was around 25–35°C and >0.98 a_*w*_ (Figure 8A). However, when the data for the two strains were combined the optimum conditions were slightly different with optimum production predicted at >30°C and >0.98 a_*w*_ (Figure 8B). For the AB10 strain and the pooled data for both strains suggests that some production will occur at around 0.91–0.90 a_*w*_ across a range of temperatures.

## Discussion

This study has examined both growth and AFB_1_ production by a group of strains of *A. flavus* isolated from pistachio nuts. This has provided useful data in relation to relative colonization and AFB_1_ production in pistachio nut-based media modified with both salt and a non-ionic solute, glycerol. This was to reflect both raw unsalted and roasted salted pistachio nuts to identify relative similarities and differences in terms of strain resilience and effects on toxin production. Changes in water availability, regardless of the solutes used had no significant differential effect on growth or indeed AFB_1_ production. The contour maps for both these treatments in relation to temperature x a_*w*_ interacting abiotic factors were similar, for both optimum and marginal conditions, using 1 or all 4 *A. flavus* strains.

Previous studies by Aldars-García et al. (2017a) examined initial germination, growth and AFB_1_ production on 3% pistachio extract medium. However, the studies were limited to 25°C and 0.85 and 0.87 a_*w*_ which were marginal for growth and toxin production. The time for germination, growth rates and time to visible colonies was estimated. They suggested that even at these a_*w*_ levels initiation of AFB_1_ production could occur within 6 days. This suggests that at 30–35°C where germination rates, growth and toxin production are much more rapid, toxin contamination might occur even earlier. Perhaps a kinetic study at 30–35°C and these a_*w*_ levels would provide useful data to predict the risks of AFB_1_ contamination. However, the present study has shown that toxin production rates were significantly higher on the milled PNA media than in raw pistachio nuts. Thus, care is needed in using such predictions of relative risks based on temperature x a_*w*_ conditions using *in vitro* studies. More data sets are required on relative AFB_1_ production by *A. flavus* strains in stored pistachio nuts to obtain more accurate predictions of risks relative to the legislative limits (Aldars-García et al., 2017b).

In the present study, *A. flavus* strains were able to grow effectively when incubated at 25–35°C, over a range of a_*w*_ levels, both *in vitro* and *in situ*. However, the range of conditions for AFB_1_ production was narrower with optimum production at 30°C in modified media, at 0.98 and 0.955 a_*w*_; and at 35°C + 0.98 a_*w*_ by strain AB3 on raw pistachio nuts. Studies by Marin et al. (2012) examined the effect of 10–30% m.c. of pistachio nuts and storage temperatures of 10–42°C and inoculated with an *A. flavus* strain. They stored the pistachio nuts for longer periods of time (up to 60 days) and used kinetic and probability models to identify growth/no growth and toxin/no toxin conditions. The probability model showed good predictions for growth/no growth after 60 days incubation. Similarly, the probability for AFs presence was predicted in 89% of the cases. They thus suggested that the EU legislative limits would be exceeded if pistachio nuts were stored for up to one-month at 20°C if the m.c. was >10–12%. The present study included relevant a_*w*_ levels equivalent to approx. 10–30% m.c. We obtained little AFB_1_ contamination at approx. ≥0.90 a_*w*_. However, our studies were limited to 10 days and longer storage periods would have perhaps provided more information on the absolute minima for growth and toxin production. However, we were able to obtain data on different strains which suggest that the overall ecological behavior was relatively similar.

In addition, it is clear that colonization rates *in vitro* were generally more rapid than that observed *in situ*. In addition, AFB_1_ production was significantly higher on the milled PNA media when compared with that produced *in situ*. This could be because of the better access to the nutrients in the milled pistachio nut-based media allowing the strains to grow faster and produce more toxins. *In situ*, the surface area is different and initial colonization and access to the nutrients may be slower or delayed. It was interesting that these strains isolated from pistachio nuts could all grow well at 35°C. Usually *A. flavus* strains from maize and peanuts are more sensitive to this temperature with optima closer to 28–30°C (Giorni et al., 2008; Abdel-Hadi et al., 2012; Bhatnagar et al., 2018). Thus, ecologically this may be important when examining intervention strategies to reduce AFs contamination of pistachio nuts during drying, storage and processing phases. In addition, the good tolerance of 35°C suggests resilience also in relation to climate-related more extreme abiotic factors (Medina et al., 2017).

Previous studies by Fernane et al. (2010a, b) showed that about 70% of *A. flavus* strains from a total of 204 were able to produce AFB_1_ and AFB_2_ on pistachio nuts. Aldars-García et al. (2015) used a predictive modeling approach to predict potential AFB_1_ contamination of pistachio nuts based on environmental conditions. They found that using this approach it was possible to predict 70–81% of samples correctly with regard to growth and in 67–81% of the cases contamination with AFB_1_ based on the models developed from their *in vitro* studies. Recently a mechanistic model has been developed using the available data on the life cycle of *A. flavus* ecologically as well as the pistachio development and the prevailing abiotic conditions (Kaminiaris et al., 2020). Ecological data on a wider number of strains isolated from pistachio nuts obtained in the present study might also be beneficial for the further development of the “AFLA-PISTACHIO” model for improving the accuracy and validation of evaluating the relative risks in different pistachio-producing countries.

## Conclusion

This study has shown that optimum interacting abiotic conditions of temperature × a_*w*_ for growth and AFB_1_ production by up to four strains of *A. flavus* isolated from pistachio nuts was very different from the ecology of strains colonizing maize and peanuts. It was interesting to note that there was no difference in growth or toxin production in PNA media modified with salt or the non-ionic solute glycerol. This also suggests that these strains have evolved to have better resilience to elevated temperatures although the water stress tolerance may well be similar. In addition, AFB_1_ production on raw pistachio nuts was much lower than on milled pistachio nut media. The contour maps clearly show the optimum and marginal conditions of these two interacting abiotic factors on both growth and toxin production. It is recommended that the safe storage m.c. for pistachio nuts is <8–10% for medium term storage under ambient temperature conditions (20–25°C). However, they are very hygroscopic because of their fatty acid content. Thus, they can easily re-adsorb moisture during bulk transport through different climatic regions and poor storage, resulting in a deterioration in quality and an increase in toxin contamination.

## Data Availability Statement

The raw data supporting the conclusions of this article will be made available by the authors, without undue reservation.

## Author Contributions

AB carried out the research work. EG-C carried out the modeling work. AM and NM supervised the research and drafted the manuscript. All authors contributed to the article and approved the submitted version.

## Conflict of Interest

The authors declare that the research was conducted in the absence of any commercial or financial relationships that could be construed as a potential conflict of interest.
