# Supplementary material for: Interacting Abiotic Factors Affect Growth and Aflatoxin B1 Production Profiles of Aspergillus flavus Strains on Pistachio-Based Matrices and Pistachio Nuts
Source: Front Microbiol. 2021 Jan 20;11:624007. doi: 10.3389/fmicb.2020.624007 (PMC7855869; doi:10.3389/fmicb.2020.624007)
Supplement: Supplementary file 1 [file Data_Sheet_1.docx]

Table 1. The oligonucleotide sequences of the primers used for confirming identification of the *A. flavus* strains isolated from pistachio nuts in Saudi Arabia.

**Primers Primer sequence Position**

ITS1 TCCGTAGGTGAACCTGCGG 1761-1779

ITS2 GCTGCGTTCTTCATCGATGC 2024-2043

ITS3 GCATCGATGAAGAACGCAGC 2024-2043

ITS4 TCCTCCGCTTATTGATATGC 2390-2409

Table 2. Sequencing results of isolated strains and type strain using ITS1 & 2 and ITS3 & 4 primer pairs for molecular identification.

**Strain ID GenBank ID Genus Species Similarity %**

**ITS1 & 2 ITS 3 & 4**

_______ _________ _________ ________ ______ ________

NRRL 3357* M1204.653 BP4 *Aspergillus flavus* 100/99

AB3 A4S3_13 SCAU-F-142 *Aspergillus flavus* 100/98

AB4 SV/09-05 UOMS28 *Aspergillus flavus* 96/99

AB5 M1204.653 M1204.653 *Aspergillus flavus* 100

AB10 M1204.653 LPSC 1183 *Aspergillus flavus* 100/99

*Type strain from the Agricultural Research Services Laboratories of the US Department of Agriculture USDA, New Orleans).
